# Nutritional Screening During Pregnancy: Women’s Views of Weight Monitoring as Part of This Process

**DOI:** 10.3390/nu17091516

**Published:** 2025-04-29

**Authors:** Catherine R. Knight-Agarwal, Lorna Munro, Stacy Morgan, Ashley Gschwend, Cassie Fekete, Michelle Minehan

**Affiliations:** Faculty of Health, The University of Canberra, 11 Kirinari Street, Bruce, Canberra, ACT 2617, Australia; lorna.munro@hotmail.com (L.M.); stacy@tmft.com.au (S.M.); gschwend.ashley@gmail.com (A.G.); cassie.fekete@gmail.com (C.F.);

**Keywords:** gestational weight gain, pregnancy nutrition, weight monitoring

## Abstract

**Background**: Worldwide, appropriate weight maintenance is one of the most important elements of human health, and this is especially true for pregnancy. Gestational weight gain below or above the recommended range is associated with numerous adverse outcomes. In addition, it may induce epigenetic changes leading to an increased risk of developing future chronic disease, such as obesity and type 2 diabetes, in both the woman and her offspring. Despite this, little is known of the views and experiences of pregnant women regarding weight monitoring and advice during the antenatal period. **Methods**: A qualitative study using individual interviews was undertaken in south-eastern Australia. Sixteen women with varying body mass index participated. Interviews were audio recorded and transcribed verbatim. Data was analyzed using interpretative phenomenological analysis. In any phenomenological study, the researcher’s objective is to elicit the participant’s views on their lived experiences. **Results**: Three major themes emerged: (1) The physiological perspective of weight monitoring during pregnancy; (2) The psychological perspective of weight monitoring during pregnancy; (3) The sociological perspective of weight monitoring during pregnancy. **Conclusions**: The findings from this study may assist the future development and support of weight monitoring information and practices for pregnant women. Women want more individualized support regarding weight monitoring during pregnancy.

## 1. Introduction

Worldwide, appropriate weight maintenance is one of the most important elements of human health, and this is especially true for pregnancy [1]. Despite this, the usefulness of weighing women during antenatal care was brought into question during the 1980s. It was revealed that maternal weight monitoring had little predictive value for the detection of various obstetric complications, such as preeclampsia, and as a result ceased to be recommended as a screening tool [1]. In addition, due to changing social attitudes, pregnant women and health professionals found the topic of ‘weight’ increasingly difficult to talk about [2].

Nevertheless, in recent years growing evidence, including the significant rise in maternal obesity [3], has precipitated emergence of an alternate viewpoint leading to the development of both Australian and international antenatal care clinical practice guidelines on gestational weight gain (GWG) [4,5,6].

GWG is defined in terms of the amount of weight a woman acquires between conception and immediately before birth. In most jurisdictions, GWG adequacy is measured using body mass index (BMI) specific ranges established by the Institute of Medicine (IOM) [6] (see Table 1). GWG below the recommended range is associated with higher risk of stillbirth [7], small-for-gestational-age (SGA) [8], and preterm delivery [8]. Conversely, GWG above the recommended range is associated with numerous short-term adverse outcomes, such as caesarean section [9], pregnancy-related hypertension [9], macrosomia and/or large-for-gestational age (LGA) [9]. It can also induce epigenetic changes leading to an increased risk of developing future chronic disease, such as obesity and type 2 diabetes, in both the woman and her offspring [10].

For all patient populations (both pregnant and non-pregnant), nutritional screening by a dietitian is the gold-standard and involves the collection, assessment and monitoring of important information including anthropometric data, e.g., body weight; biochemical indices, e.g., iron status; clinical variables, e.g., co-morbidity such as obesity; and diet, e.g., current intake. Traditionally, dietitians’ primary involvement in antenatal care has been in the multidisciplinary treatment of gestational diabetes mellitus (GDM). Nevertheless, outside of GDM management, many Australian women have limited access to dietetic support during pregnancy [11].

In the Australian public hospital system, a woman’s initial antenatal visit is overseen by either a midwife or an obstetrician. This involves a comprehensive clinical and psycho-social assessment that should include elements of nutritional screening, such as weight measurement and monitoring, in addition to general advice on nutrition and physical activity [12]. As such, midwives and obstetricians play an important role in supporting pregnant women to adopt and maintain healthy lifestyle behaviors, especially if dietitian access is limited [13]. However, gaps exist between current guidelines and clinical practice with evidence accruing that GWG monitoring and healthy lifestyle counselling are not being provided consistently to women during pregnancy despite both local and international recommendations [14,15,16,17].

Therefore, the aim of this study was to use Interpretative Phenomenological Analysis (IPA) to explore the views and experiences of pregnant women in the Australian Capital Territory (ACT) regarding weight monitoring during pregnancy.

## 2. Methods

### 2.1. Study Design

The researchers consider reality to be socially constructed and aim to produce subjective findings via inductive reasoning [18]. Rather than setting up a series of hypotheses, the research presented here was guided by a semi-structured interview guide that focuses on the examination of experience suggesting a phenomenological course of enquiry is appropriate. IPA is concerned with individuals’ lived experience and how they make sense of that experience [18,19].

### 2.2. Participants and Recruitment

Purposive sampling requires that individuals be deliberately selected with an explicit purpose in mind, namely, to address the research aim and because they are rich sources of data in relation to this. The participants in our study were selected based on their personal characteristics (e.g., pregnancy), their experience of a specific issue (e.g., gestational weight) and their behavior (e.g., gestational weight monitoring). Printed flyers, social media and the University of Canberra staff intranet were used to advertise the study. According to Smith et al., an adequate sample size is one that sufficiently answers the research question, the goal being to obtain cases rich in relation to this [20]. Therefore, sixteen participants were deemed an adequate number, particularly as saturation was achieved following the last interview. Bracketing was not considered necessary, as Heideggerian phenomenology supports that it is impossible to negate human experiences related to the phenomenon under study resulting in personal awareness being intrinsic to phenomenological research [20]. As such, reflexivity was practiced by maintaining an open dialogue, among the entire study team, throughout the research process [18,19,20]. Women who agreed to be interviewed provided signed consent, with each given a unique number to ensure anonymity. Semi-structured interviews were arranged with pregnant women aged 18 years or over, more than or equal to 12 weeks gestation with varying weight status.

### 2.3. Materials and Procedure

Once our study team identified the primary research question, the next step was to collaboratively create the interview guide. A review of the published literature [1,5,6,7,8] was undertaken to support this process, in addition to discussions around the type of questions that should be asked and how they should be phrased, particularly as it was acknowledged that weight can be a sensitive issue to talk about. The team included researchers who had experienced pregnancy and various levels of GWG and/or monitoring in addition to those who had not. All researchers had a strong background in nutrition, with the lead researchers M.M and C.R.K.A having worked previously with women in pregnancy. This holistic approach to guiding development reduced the chance of unintentional biases in the interview questions. To ensure a standardized interview process was followed, all interviewers underwent training with the study’s lead investigators (C.R.K.A and M.M) who are experienced qualitative researchers. As weight can be a sensitive topic to discuss, questions were kept open, allowing participants to talk candidly with minimal interruption. Interviewers (S.M., A.G., L.M., C.F.) were mindful to use non-judgmental language, and speaking with participants separately, as opposed to being part of a group discussion, helped to support free expression. It also aided in establishing rapport between the participant and the interviewer. Participants were asked to choose a time and place that suited their schedules for the face-to-face interviews. Interviews lasted on average 45 min and were audio-recorded and transcribed verbatim into a word document.

### 2.4. Ethical Approval

Ethical approval to conduct this research was received from the University of Canberra Human Research Ethics Committee (HREC ref 17135).

### 2.5. Data Analysis

IPA is a popular methodological framework to use in studies of this kind. The IPA protocol described by Biggerstaff and Thompson (2008) was followed to assess the data [19]. Transcriptions were produced without the use of computer software to facilitate fidelity and interpretation. Data analysis included (1) close, line-by-line coding of the experiential claims and understandings of each participant; (2) identification of common patterns in the experiential material; and (3) a dialogue between the researchers and data about what it might mean for participants to have concerns, in a particular context, leading to the development of a more interpretative account [20]. When this process had been repeated with each transcript, the resulting set of preliminary themes was examined to identify recurrent patterns across all transcripts. Three superordinate themes were decided on and corresponding quotes assigned to each of these. Quotes used were given unique classifiers to firstly identify the participant and secondly the location within the relevant transcript. For example, P3L72 indicates the quote came from participant 3 and begins on line 72 of the transcript. This process was undertaken with the entire study team as an important form of triangulation.

## 3. Results

Sixteen women participated in the one-on-one semi-structured interviews (see Table 2).

Participants had varying opinions about weight monitoring during pregnancy and conflicting ideals regarding its use in antenatal care. Three themes were identified: (1) The physiological perspective of weight monitoring during pregnancy; (2) The psychological perspective of weight monitoring during pregnancy; (3) The sociological perspective of weight monitoring during pregnancy. Results are described under these themes with participants’ quotes to support findings.

### 3.1. The Physiological Perspective of Weight Monitoring During Pregnancy

It was believed that weight monitoring had the potential to influence both a woman and her child’s short- and long-term physiological health: “during and after the pregnancy, I don’t want to put on too much weight where it’s not safe for myself or the little one” (P11, L235). Some women were particularly mindful of weight monitoring due to the experiences relayed to them by others: “So, my mum is quite overweight, and I’ve always been told, well you only get overweight if you don’t pay attention to what’s going on” (P3, L48).

Each woman is an individual and, as such, will experience different physiological responses during pregnancy: Although weight hugely fluctuates, from woman to woman,… having those (weight gain) stats is only going to help…” (P13, L40). Women intimated there was a risk, for some, that weight may get out of control without regular monitoring: “It is a useful indicator… especially with other measures… (because) if between two appointments you go up ten kilos and the baby has only grown by a couple of centimeters… (that could have detrimental consequences)” (P14, L65).

Like other types of clinical monitoring during pregnancy, many women expressed the belief that weighing should be a normal part of maternity care: “when someone actually weighs you it helps you keep on track to achieve your weight gain recommendation” (P1, L74). In addition, it was acknowledged by some women that calculation of pre-pregnancy BMI was an important indicator of nutritional status: “to identify if you are (over nourished) or if you are malnourished” (P16, L188).

Regular weighing and conversations around this issue were viewed by many as an important form of knowledge acquisition. One woman posed the following hypothetical question: “Should you be putting weight on in your first trimester?… should you be putting weight on in your second trimester?…, like what does (it all) mean? And being clear about your uterus weighs this, your baby weighs this… ” (P9, L46). Another woman went on to point out: “I was really surprised that my doctor didn’t weigh me. It really bothered me… pregnant women are highly concerned for their babies and want to do what’s right by them, no information goes unwanted” (P9, L32). It was recognized that discussions about weight, regardless of who initiates them, may trigger an important dialogue with relevant health professionals: “If there is something amiss with your weight… the next step is how do you manage that issue? I think it’s really important because you don’t know what you don’t know” (P16, L199).

### 3.2. The Psychological Perspective of Weight Management During Pregnancy

It was acknowledged that weight monitoring could be a ‘potentially’ stressful exercise for some, as one woman commented: “Personally, I think it can create more emotional damage and spiral women into negative thinking… that can possibly have an (adverse) effect on eating” (P5, L80). On the flip side, others believed that a pregnant woman’s psychological wellbeing may be adversely affected by not partaking in routine weighing: “If after you’ve… given birth and you’ve found out you’ve put on 10 kilos too much and that could’ve (been) avoided, I feel like your mental health is probably worse off” (P8, L165).

Acknowledgment was made that even though weight can be a sensitive issue it was still a salient topic for discussion: “I think it’s important for health professionals to say something instead of pussyfooting around… because then you end up with people like my mum who struggled with weight for the rest of her life and she blames it on pregnancy” (P8, L247). Likewise, others expressed the belief that it is best to face the issue of weight rather than pretend it does not exist: “I’d rather know (how my weight is tracking) than stick my head in the sand” (P3, L48).

Participants believed that pregnant women are often wrapped in ‘cotton wool’ with weighing being a taboo topic in many instances: “I think people are so aware of, like, I can’t insult you and don’t want to make you feel bad about yourself especially when you’re full of hormones” (P9, L76).

Some participants spoke about being weighed as part of their antenatal care despite having no conversations initiated by health professionals around why they were weighed or what the scales reported: “they write it down and we don’t talk about it at all” (P8, L149). The lack of opportunity to openly speak about these issues was upsetting for many: “I know for all of the appointments I have had, weight and weighing isn’t discussed, it’s not even an option for discussion” (P14, L72). Feeling unsupported by health professionals created negative emotions in some women: “I’ve gotten out of control… I’ve put on 15 kg… for the whole pregnancy… they only weighed me at the first appointment but not since” (P16, L310).

Confusion was expressed regarding the lack of uniform weight monitoring practice that women had observed between different health professionals: “I’ve got friends that go to this doctor, so I’d be like how come I got weighed and she didn’t?” (P3, L160). Other’s stated that their maternity care providers had communicated the belief that weight monitoring throughout pregnancy may invoke unnecessary anxiety and, as such, should be avoided: “I was actually told not to weigh myself… they said it’s just going to stress you out so don’t worry about it.” (P11, L252).

Ultimately, it was acknowledged that pregnant women are unique beings with individual needs and wants. The suggestion was made that: “If people get upset by (weighing in pregnancy) then maybe it can be an opt-in and opt-out measure…” (P14, L72).

### 3.3. The Sociological Perspective of Weight Monitoring During Pregnancy

The societal myth that pregnancy requires a significant increase in energy requirements was communicated by many: “I think for women… regular weighing is an issue that should be raised because you’re so much more prone to go ‘oh well I’m eating for two now and stack on the weight’” (P9, L72). Similar sentiments were expressed by another participant who felt that because “I’m pregnant it’s okay not to worry about what I monitor, or (whether I) gain weight” (P12, L370).

Despite some women being more relaxed regarding weight monitoring during the antenatal period, this was juxtaposed by the uneasiness many felt about their changing body shape. One participant made the comment: “The things surrounding weight that have affected me most is comparing myself to other pregnant women who are at the same gestational week as me through hashtags” (P10, L207).

Women made the point that the general public seem to believe it is acceptable to comment on a pregnant woman’s weight even though they probably would not do so with a non-pregnant woman. This led to feelings of anxiety in some, with one woman expressing upset having been told “how big I am on social media” (P10, L207).

Several participants undertook their own weight monitoring due to the fact they were not weighed as part of routine antenatal care. Despite recognising that it is both normal and natural to put on weight during pregnancy, some women were unprepared for the reality: “oh my god” I’ve never seen these numbers (on the scales). These are numbers that I never imagined…” (P3, L166). The ‘shock’ led them to seek advice not from their maternity care provider but via social media to “see what a normal weight gain during pregnancy should be” (P5, L125). While others admitted turning to ‘mummy blogs’ and ‘Facebook groups’ (where)“there’s a lot of (women)… concerned about their weight gain, like I’m such and such weeks and I’ve already put on so much weight… how do I stop?” (P10, L155).

Ultimately, participants in our study desired a safe and reliable space to discuss weight monitoring with their maternity care provider with the following comment summing up this sentiment: “I would have liked it If I didn’t have to Google to find out if I put on the right amount or not” (P8, L179).

From a broader societal perspective, many women felt that weight monitoring during pregnancy was particularly important due to: “the world-wide obesity issues” and “how preventing significant weight gain in pregnancy may help” (P9, L22).

## 4. Discussion

For women, the transition from pregnancy to motherhood is a critical period of social, psychological, and physiological change. Pregnancy has been described as a ‘teachable moment’ for the promotion of healthy lifestyle behaviors, including weight management, as most women are concerned about the wellbeing of their unborn child in addition to being in frequent contact with antenatal healthcare professionals [21]. A recent systematic review and meta-analysis reported that compared with routine antenatal care, dietary interventions embedded within maternity services to reduce GWG were associated with a reduced risk of gestational diabetes, preterm delivery, LGA, and neonatal intensive care admission [22]. Despite evidence such as this, several participants in our study reported that weight monitoring and advice about GWG was inconsistent or non-existent. Previous research has identified several barriers midwives and obstetricians face regarding their support of pregnant women to adopt healthier behaviors, including lack of training, poor confidence, time pressures and concerns about damaging the patient–provider relationship [21,23]. Of note is an Australian-focused literature review reporting that accreditation standards for nursing and midwifery courses provide no content on nutrition [24]. Likewise, in the Royal Australian New Zealand College of Obstetricians and Gynecologists (RANZCOG) Integrated Training Program (which includes curriculum for registrars who are preparing for fellowship of the college), there is no specific module for nutrition or dietary assessment [25]. Consequently, midwives and obstetricians may find benefit in collaborating with dietitians in the development and implementation of local pregnancy nutrition best practice guidelines which include weight monitoring guidance. Additionally, a 2020 Australian study mapped preconception, antenatal and postnatal dietetic service delivery across various hospital settings. Low staffing levels were reported with major concerns identified about the lack of capacity to provide evidence-based nutrition care [26]. The authors concluded by advocating for more dietitians in maternity services across Australia [26].

Most participants in our study expected to be routinely weighed throughout their pregnancy and were surprised when this did not occur. Furthermore, they identified several potential benefits to weight monitoring, such as providing reassurance and helping to minimize postpartum weight retention. Likewise, a UK feasibility study found that women were more motivated to think about their GWG in terms of dietary habits and physical activity because of being weighed and responded positively to the practice. The midwives reported no issues in incorporating routine weighing into antenatal care and believed women were glad to have their weight routinely monitored [27]. Similar results were reported by the authors of an Irish qualitative study investigating the perceptions of women regarding weighing during pregnancy [28]. Current evidence suggests greater consistency regarding weight monitoring advice and activities is needed.

Participants in our study acknowledged that weight can be a sensitive issue. Nevertheless, they still believed it was a salient topic for discussion. An Australian randomized controlled trial compared routine weight monitoring to usual care during pregnancy. The majority (73%) of women reported being very satisfied with the practice in addition to claiming they felt no concern around being weighed [29]. Previously, clinicians have claimed that routine weighing may create un-necessary stress, particularly in those women with a high pre-pregnancy BMI [30,31,32]. However, a recent study demonstrated that excessive GWG in uncomplicated pregnancy is a warning sign of anhedonia and anxiety, whereas inadequate GWG was found to be a significant indicator of depression [30]. Such findings add to the increasing body of evidence that for many women monitoring of and preventing inappropriate GWG may prevent adverse psychological symptoms as opposed to being the cause of them [29,30]. We acknowledge that several studies have reported that routine weighing during pregnancy is stressful for some women, particularly those with a high pre-pregnancy BMI [31,32]. Additional training by antenatal healthcare providers that are not dietitians to obtain skills necessary to relay GWG information and facilitate regular weight monitoring in a non-judgmental and informative way is warranted.

Participants in our study expressed an increased awareness of the importance of nutrition during pregnancy, including weight monitoring and appropriate GWG. However, the amount and type of nutrition-related information provided by health professionals varied considerably, an issue that has been reported elsewhere [23,33]. When participants were not able to obtain the information and support they desired from their antenatal healthcare provider, it was sought elsewhere, for example, the internet. A recent systematic review investigated the impact of social media influences on pregnancy, birth, and early parenting experiences. The authors reported that the most common reason for pregnant women to access information via this medium was uncertainty [34]. Many women are vulnerable during pregnancy, as they navigate considerable change, and this is particularly true regarding the uptake of information and, more concerningly, misinformation. Misleading narratives about nutrition-related information, such as weight monitoring, pose a challenge to clinicians by undermining medical trust whilst also contributing to poorer outcomes for both women and their offspring. Antenatal care facilities are in a prime position to support women to access evidence-based, nutrition-related information, such as credible weight monitoring practices and appropriate GWG advice, via a multitude of different sources, including not only themselves but social media as well.

### Strengths and Limitations

In terms of strengths, we gave a voice to women who had experienced various weight management practices during pregnancy in addition to using rigorous qualitative methods during all stages of this research. Regarding limitations, the sample size was small, although it was considered adequate according to qualitative experts. We also acknowledge that a clinician’s approach to discussing GWG and weight monitoring may be dependent on a woman’s BMI. Likewise, this is something we could explore in more detail if the sample of women interviewed (either underweight or healthy weight or overweight) were even more homogenous than the sample presented within our research. In addition, we acknowledge that the recruitment strategy may have been potentially biased to women interested in weight management. Collection of additional demographic data, such as level of education and socioeconomic status, would have provided further context, particularly in relation to the synthesis of our results and ensuing discussion. Future research of this kind would be strengthened by including more demographic data. We also acknowledge that the views expressed here may not be representative of other pregnant women in Australia. It is, therefore, important to emphasize that the aim of this study was to explore the views and experiences of participants in relation to weight monitoring during pregnancy and was not intended to be an evaluation of current antenatal practice.

## 5. Conclusions

Participants from our study expressed a desire for more individualized support regarding weight monitoring and GWG advice during pregnancy. Ongoing collaboration between midwifery, obstetric and dietetic professionals is paramount if these needs are to be met. Extra resources in terms of staffing, specialized training and funding are required to achieve this.

## Figures and Tables

**Table 1:** IOM GWG guidelines [6].

Pre-Pregnancy BMI Category	Recommended Weight Gain (kg)
Underweight (<18.5)	12.5–18
Normal weight (18.5–24.9)	11.5–16
Overweight (25–29.9)	7–11.5
Obese (≥30)	5–9

**Table 2:** Demographic information of study participants.

Participant	Gestation (Weeks)	Pre-Pregnancy Height (m)	Pre-Pregnancy Weight (kg)	Pre-Pregnancy BMI (kg/m^2^)	BMI Class
1	39	1.66	96	34.8	Obese I
2	20	1.78	67	21.1	Normal range
3	23	1.73	77	25.7	Overweight
4	37	1.67	94	33.7	Obese I
5	35	1.71	66	22.6	Normal range
6	38	1.58	58	23.2	Normal range
7	18	1.74	91	30.1	Obese I
8	16	1.72	90	30.4	Obese I
9	19.5	1.68	68.8	24.3	Normal range
10	20	1.73	81	27	Overweight
11	23	1.68	59	20.9	Normal range
12	26	1.68	64	22.6	Normal range
13	21	1.71	92	31.5	Obese I
14	28	1.70	68	23.5	Normal range
15	15	1.65	57	20.9	Normal range
16	18	1.70	20	17.3	Underweight

## Data Availability

We agree to make all data available on request from the corresponding authors.
